# Enhancing HIV/AIDS knowledge and attitudes through a healthcare-school collaborative model: a study among Shanghai high school students

**DOI:** 10.1186/s12981-025-00805-w

**Published:** 2025-11-11

**Authors:** Peipei Wang, Xiaojing Yu, Bin Zhang

**Affiliations:** 1 Hongkou District Jiaxing Road Subdistrict Community Health Service Center, Shanghai, 200080 China; 2 Yangpu District Health Promotion Center, Shanghai, 200082 China

**Keywords:** Medical school alliance, New model, AIDS, Cognitive attitude

## Abstract

**Background:**

To evaluate the effectiveness of a healthcare-school collaborative intervention model in improving HIV/AIDS-related knowledge and attitudes among high school students.

**Methods:**

A randomized controlled trial was conducted with 444 Grade 10–11 students from a Shanghai high school. Participants were cluster-randomized into two groups: a traditional model group (*n* = 299, routine health education) and a new model intervention group (*n* = 145, receiving healthcare-school collaborative interventions, including on-campus clinics, peer education, and digital outreach). Self-administered questionnaires measured outcomes at baseline and after the intervention.

**Results:**

Baseline data revealed high awareness of HIV transmission routes (91.6%) but lower understanding of non-transmission routes (75.88%). Post-intervention, the new model group demonstrated significantly higher knowledge rates than the traditional group in critical areas: *recognizing that HIV is not transmitted through dining together* (92.91% vs. 85.71%), *mosquito bites* (90.55% vs. 34.59%), *mother-to-child transmission* (96.85% vs. 89.85%), and *condom effectiveness* (85.04% vs. 75.19%) (all *P* < 0.05). Furthermore, 87.4% of students in the new model group expressed willingness to maintain friendships with peers living with HIV/AIDS, surpassing the traditional group (78.57%, *P* = 0.035). Knowledge scores were significantly higher in the new model group (9.28 ± 1.27 vs. 8.46 ± 1.27, *P* < 0.05). Satisfaction surveys showed high approval for the intervention, with 89.76% of participants satisfied with the format and 83.46% perceiving it as useful.

**Conclusion:**

The healthcare-school collaborative model, integrating on-site health services, multimedia education, and behavioral guidance, effectively enhances HIV/AIDS knowledge, reduces stigma, and fosters healthy sexual attitudes among adolescents. This model offers a scalable and effective framework for school-based HIV prevention efforts.

## Introduction

AIDS has consistently ranked as the second-leading cause of infectious disease-related mortality in this age group, with high school and college students exhibiting the highest incidence rates of HIV infection [1–5]. In recent years, over 3,000 young students have been newly infected with HIV annually in China [6]. High school represents a crucial period in adolescent physical and mental development. High school students are considered a high-risk population for HIV infection due to limited sexual health education, immature cognitive development, and susceptibility to peer pressure and misleading online content [7–9].

Effective HIV/AIDS education in schools is essential for improving students’ awareness and ability to prevent infection [10]. In 2024, China launched the National HIV/AIDS Prevention and Control Plan (2024–2030), which specifically identifies schools as key settings for strengthening health literacy and promoting HIV self-testing. The plan aims to raise HIV awareness to over 90% among the general population and over 95% among key populations by 2025. Despite these policy efforts, challenges persist at the local implementation level. In many universities, student participation in health programs remains low, health education relies on outdated materials, and there is a lack of integration between campus-based and community-based health services [11, 12]. These issues highlight the need for more engaging, accessible, and evidence-based educational models.

To address these issues, this study employs a project-based research approach, integrating healthcare and educational resources to develop and evaluate a novel collaborative intervention model between healthcare institutions and schools. This model emphasizes proactive prevention, aiming to curb HIV transmission before it occurs through comprehensive health education and accessible support services. By enhancing awareness, fostering accurate understanding of HIV/AIDS, and promoting practical prevention skills, the intervention seeks to encourage adolescents to adopt healthy sexual behaviors and reduce stigma. The outcomes of this study are expected to inform the development of more effective, scalable school-based HIV prevention strategies and contribute to the advancement of comprehensive sexual health education in China.

## Methods

### Study participants and grouping procedure

The study enrolled Grade 10 (equivalent to Chinese senior high first year) and Grade 11 (senior high second year) students from a high school in Shanghai. A cluster-randomized sampling design was implemented, randomly assigning 4 out of 12 classes (Class 2 and 3 of Grade 10; Class 4 and 5 of Grade 11) to the new model intervention group, with the remaining classes serving as the traditional model group (control group). Ethical approval for this research was obtained from the Ethics Committee of Shanghai Fourth People’s Hospital (Approval No. 2023122-001).

### Survey methods and content

A self-designed questionnaire was developed based on literature review and the latest global progress in AIDS prevention [13, 14]. The questionnaire covered demographic characteristics, AIDS-related knowledge and attitudes, and satisfaction evaluations of intervention activities. Both groups independently completed anonymous paper-based surveys during breaks. The questionnaire demonstrated good measurement properties, with a content validity index (CVI) of 0.87 based on expert review. Internal consistency was verified in a pilot test with 30 students, yielding Cronbach’s alpha values of 0.80 (knowledge items) and 0.74 (attitude items).

### Intervention measures

This community-based intervention trial included baseline surveys conducted in February 2024, followed by a 3-month intervention period, with final surveys completed in June 2024.

The students in the new model group received a multi-component HIV education program combining clinical, group, and digital strategies. A professional team from community hospitals established an on-campus HIV self-testing counseling clinic. A total of 108 students (74.48%) received at least one face-to-face consultation. Three in-person lectures (one per month) were delivered, covering topics such as HIV transmission, self-protection, and stigma reduction. The average attendance rate was 93.1%. During non-clinic hours, health education was delivered via new media platforms (e.g., WeChat and QQ for consultations, weekly HIV science updates via official accounts). Articles garnered an average of ~ 370 views per post, with over 4,400 cumulative reads during the intervention period. Defined as attending ≥ 1 consultation, ≥ 1 group lecture, and reading ≥ 1 online post. 79 students (54.48%) met this criterion.

The students in the traditional model group received monthly routine health education delivered by school staff, including printed pamphlets and general sexual health information. The average attendance rate for routine sessions was 88.6%, slightly lower than the 92.5% in the intervention group.

### Statistical analysis

Data were entered into Excel and analyzed using SPSS 22.0. Continuous variables were described as mean ± standard deviation (SD), with intergroup comparisons using *t*-tests. Categorical variables were described as frequencies and percentages, analyzed via *chi*-square (*χ²*) tests. The significance level was set at *α* = 0.05.

## Results

### Basic information

A total of 444 students were surveyed, including 145 in the new model group and 299 in the traditional model group, with an average age of 16.3 years, ranging from 15 to 17 years. The gender distribution was nearly balanced, with a male-to-female ratio close to 1:1. A total of 888 questionnaires were distributed before and after the intervention, with 837 valid responses collected, yielding an effective response rate of 94.26%.

### Comparison of AIDS-related knowledge before and after intervention

Post-intervention analysis revealed that the new model group demonstrated statistically significant improvements in awareness rates compared to the traditional model group across multiple domains (Table 1). Specifically, 92.91% of participants in the new model group recognized that shared dining does not facilitate HIV transmission, surpassing the 85.71% observed in the traditional group. Similarly, awareness that mosquito bites cannot transmit HIV rose to 90.55% versus 34.59%, while understanding of mother-to-child transmission during pregnancy reached 96.85% compared to 89.85%. Additionally, 85.04% of the intervention group acknowledged the risk-reduction benefits of proper condom use, exceeding the traditional groups 75.19%. While the overall HIV-related knowledge scores significantly increased in the intervention group after the 3-month program, minor declines were observed in a few specific items. For example, the correct response rate for “Sexual intercourse transmits HIV” decreased slightly from 99.31% at baseline to 98.43% at follow-up, and for “Blood transfusion transmits HIV” from 99.31% to 95.28%. These exceptions were not statistically significant (*p* > 0.05), and may reflect sampling variation or external factors.

**Table 1:** Comparison of HIV/AIDS-related knowledge awareness rates among high school students in Shanghai [n (%)]

Survey item	Baseline survey				Final survey			
	New model group (*n* = 145)	Traditional model group (*n* = 299)	χ²	*P*	New model group (*n* = 127)	Traditional model group (*n* = 266)	χ²	*P*
Transmission routes								
Sexual intercourse transmits HIV	144 (99.31)	292 (97.66)	0.717	0.397	125 (98.43)	259 (97.37)	0.087	0.768
Blood transfusion transmits HIV	144 (99.31)	292 (97.66)	0.717	0.397	121 (95.28)	261 (98.12)	1.618	0.203
Sharing syringes transmits HIV	142 (97.93)	293 (97.99)	0.000	1.000	122 (96.06)	263 (98.87)	2.139	0.144
Mother-to-child transmission	128 (88.28)	274 (91.64)	1.289	0.256	123 (96.85)	239 (89.85)	5.798	0.016
Correct condom use reduces risk	106 (73.10)	218 (72.91)	0.002	0.966	108 (85.04)	200 (75.19)	4.922	0.027
Non-Transmission routes								
Handshakes/hugs do not transmit HIV	142 (97.93)	291 (97.32)	0.004	0.952	122 (96.06)	260 (97.74)	0.382	0.536
Dining together does not transmit HIV	111 (76.55)	249 (83.28)	2.880	0.090	118 (92.91)	228 (85.71)	4.231	0.040
Mosquito bites do not transmit HIV	57 (39.31)	114 (38.13)	0.058	0.810	115 (90.55)	92 (34.59)	108.000	< 0.001
Studying/living together does not transmit HIV	134 (92.41)	270 (90.30)	0.532	0.466	117 (92.13)	240 (90.23)	0.373	0.541
Coughing/sneezing does not transmit HIV	99 (68.28)	225 (75.25)	2.409	0.121	108 (85.04)	209 (78.57)	2.305	0.129

### AIDS-related attitudes before and after intervention

The analysis revealed persistent stigma among high school students, with 45.52% of the new model group and 52.84% of the traditional model group expressing willingness to dine with individuals living with HIV. Following the intervention, a significantly higher proportion of students in the new model group reported intent to maintain friendships with classmates diagnosed with AIDS, at 87.4% compared to 78.57% in the traditional model group (Table 2), achieving statistical significance at *P* < 0.05. Within the new model group, mean attitude scores increased from 4.78 to 5.40 (*P* < 0.05), indicating a modest internal improvement.

**Table 2:** Comparison of HIV/AIDS-related attitudes among high school students in Shanghai [n (%)]

Survey item	Baseline survey				Final survey			
	New model group (*n* = 145)	Traditional model group (*n* = 299)	χ²	*P*	New model group (*n* = 127)	Traditional model group (*n* = 266)	χ²	*P*
People living with AIDS should not be isolated	101 (69.66)	197 (65.89)	0.628	0.428	102 (80.31)	194 (72.93)	2.520	0.112
People with AIDS are not a societal burden	106 (73.10)	230 (76.92)	0.774	0.379	97 (76.38)	206 (77.44)	0.055	0.814
Would remain friends with classmates living with AIDS	101 (69.66)	227 (75.92)	1.986	0.159	111 (87.40)	209 (78.57)	4.431	0.035
Would seek help and treatment if infected	131 (90.34)	272 (90.97)	0.046	0.831	119 (93.70)	245 (92.11)	0.320	0.572
Willing to dine with people living with AIDS	66 (45.52)	158 (52.84)	2.096	0.148	84 (66.14)	160 (60.15)	1.311	0.252
Willing to share classes/desks with people living with AIDS	77 (53.10)	182 (60.87)	2.423	0.120	83 (65.35)	172 (64.66)	0.018	0.893
Disapprove of premarital sexual activity	111 (76.55)	226 (75.59)	0.050	0.823	90 (70.87)	198 (74.44)	0.560	0.454

### Comparison of AIDS-related knowledge and attitude scores

A scoring system was applied to assess AIDS-related knowledge (1 point per correct answer, 0 for incorrect) and attitudes (1 point for positive attitudes, 0 for negative attitudes). After 3 months of intervention, the new model group achieved significantly higher knowledge scores (9.28 ± 1.27) compared to the traditional model group (8.46 ± 1.27) (Table 3), with a statistically significant difference (*P* < 0.05).

**Table 3:** Comparison of HIV/AIDS-related knowledge and attitude scores among high school students in Shanghai

Assessment domain	Baseline survey				Final survey			
	New model group (*n* = 145)	Traditional model group (*n* = 299)	χ²	*P*	New model group (*n* = 127)	Traditional model group (*n* = 266)	χ²	*P*
Knowledge score	8.32 ± 1.22	8.42 ± 1.31	0.749	0.455	9.28 ± 1.27	8.46 ± 1.27	-5.988	< 0.001
Attitude Score	4.78 ± 1.96	4.99 ± 1.74	1.146	0.252	5.40 ± 1.87	5.20 ± 1.81	-1.007	0.314

### Evaluation of knowledge sources and intervention satisfaction

Satisfaction evaluations demonstrated that 89.76% of participants expressed satisfaction with the intervention formats while 75.59% found the content engaging and 83.46% perceived the activities as helpful (Table 4), emphasizing the effective integration of theoretical and practical components.

**Table 4:** Evaluation of satisfaction with HIV/AIDS intervention services among high school students in Shanghai [n (%)]

Survey item	Yes	Partial	No
Satisfaction with intervention formats	114 (89.76)	8 (6.30)	5 (3.94)
Interest in intervention content	96 (75.59)	17 (13.39)	14 (11.02)
Perceived helpfulness of intervention content	106 (83.46)	14 (11.02)	7 (5.51)
Integration of theoretical and practical components	106 (83.46)	9 (7.09)	12 (9.45)

## Discussion

The survey results indicate that high school students demonstrated higher awareness of HIV transmission routes compared to non-transmission routes. For instance, in this study and similar prior research, awareness rates regarding “mosquito bites do not transmit HIV” remained low among high school and college students [15]. This highlights the need to prioritize education on non-transmission pathways in future school-based HIV/AIDS health programs to enhance students’ comprehensive understanding and reduce campus transmission risks.

Furthermore, the persistently high prevalence of negative and discriminatory attitudes toward people living with HIV (nearly half of the respondents expressed such views) highlights the urgent need to address stigma, as it poses significant societal risks. Efforts to reduce stigmatization must be central to any educational initiative aimed at effective HIV prevention.

After three months of intervention, the new model group exhibited significantly higher HIV/AIDS-related knowledge scores than the traditional model group (*P* < 0.05). While the intervention group showed slightly higher post-intervention attitude scores, the difference compared to the control group was not statistically significant, suggesting that both models had a comparable effect in the short term. This discrepancy suggests that diverse intervention activities effectively enhanced knowledge acquisition, but the transient nature of existing school-based efforts, typically confined to World AIDS Day campaigns, may hinder the translation of knowledge into sustained positive attitudes, a process requiring prolonged and multifaceted engagement [16]. Thus, systematic and sustained HIV/AIDS education is critical for adolescents [17, 18]. Future programs should emphasize fostering tolerance and empathy toward people living with HIV, enabling students to apply their knowledge in real-world contexts and disseminate socially responsible attitudes.

Interestingly, we noted that a few knowledge items in the intervention group showed a slight decline in correct response rate at follow-up compared to baseline, despite the overall improvement in total knowledge scores. For instance, items such as “Sexual intercourse transmits HIV” and “Blood transfusion transmits HIV” had baseline correct rates above 99%, which slightly dropped at follow-up. This phenomenon may be attributed to two factors. First, the number of valid follow-up questionnaires was lower than at baseline, potentially introducing sampling bias or fluctuations in item-level results. Second, during the intervention period, students were exposed to a variety of online content, some of which may have conveyed inconsistent or misleading information regarding HIV transmission. Such multimedia interference may have diluted the educational effect of our structured intervention for certain items. These observations underscore the importance of reinforcing key messages through repeated and multi-channel delivery while ensuring content accuracy and consistency across platforms. They also highlight the need for more stable follow-up measures and continuous engagement to sustain knowledge gains over time. Nevertheless, the overall trend strongly supports the effectiveness of the intervention.

While satisfaction evaluations for the new model group revealed high approval rates for intervention formats (89.76%), a minority of students expressed disinterest in the content. This calls for strategic adjustments, such as incorporating student feedback to tailor activities to their needs and diversifying engagement methods. Our satisfaction rate (89.76%) exceeds those reported in similar interventions in African settings, which may be attributed to the interactive nature of our program and the convenience of on-campus services [19, 20]. High school students, characterized by curiosity and susceptibility to external influences, primarily rely on the internet and school-based education for information [21]. However, limited smartphone access and unreliable online content pose risks of misinformation. Extensive studies confirm the pivotal role of school-based comprehensive sex education in improving HIV-related knowledge and establishing a foundation for effective prevention [11, 22]. Integrating these insights with innovative models like the DREAMS intervention [23] and Red Carpet Program (RCP) [24, 25] represents a promising direction for future research and practice.

Additionally, He et al. found that both schoolteachers and parents support the establishment of adolescent-friendly clinics within healthcare institutions, advocating for more professional, youth-oriented services [26]. As high school students transition to adulthood, fostering healthy sexual norms and prioritizing safe-sex training are essential to bridging the gap between knowledge and behavior [27, 28]. This study’s on-campus HIV self-testing counseling clinics, face-to-face consultations, and streamlined referral mechanisms improved service accessibility and expertise. Complemented by new media platforms for private consultations, the healthcare-school collaborative intervention model addressed limitations of traditional approaches (such as lack of privacy and convenience), effectively enhancing knowledge retention and attitude improvement while ensuring scalability. Notably, students reported obtaining HIV-related knowledge mainly through the internet and school-based education, highlighting the importance of optimizing digital and institutional channels for adolescent health communication.

This study has several limitations. First, the intervention was conducted in a single high school in Shanghai, which may limit generalizability to other regions or educational contexts. Second, the sample size (*n* = 444) was modest and may have limited the power to detect subtle subgroup differences. Third, the intervention period lasted only three months, and no follow-up assessment was conducted to evaluate the durability of knowledge or attitude improvements. Fourth, the use of self-reported questionnaires may have introduced social desirability bias. Fifth, due to ethical constraints, we did not collect information on parental education or occupation, which limited our ability to analyze the influence of socioeconomic factors. Future research should aim to expand the sample size, extend the duration of interventions, and incorporate routine, longitudinal education to provide deeper insights and sustained impact.

In conclusion, the healthcare-school collaborative intervention model, combining on-campus health services with multimedia engagement, significantly improved HIV/AIDS awareness, prevention knowledge, and promoted healthier sexual attitudes among high school students. This was achieved through a combination of information dissemination, cognitive education, and behavioral guidance. The high satisfaction rate among students further supports the feasibility and acceptability of this model. As adolescents remain a key population in HIV prevention efforts, school-based programs that combine clinical support with innovative, student-centered education offer a promising path forward. Future research should focus on expanding the scope, duration, and adaptability of such models to ensure long-term impact and broader applicability.

## Data Availability

The datasets generated during and/or analyzed in the current study are not publicly available due to privacy and ethical restrictions, but are available from the corresponding author on reasonable request and with approval from the Ethics Committee of Shanghai Fourth People’s Hospital.
